# Metabolomics-constrained modelling reveals dominant oxidative metabolism in the Egyptian fruit bat myocardium

**DOI:** 10.1371/journal.pone.0349571

**Published:** 2026-06-02

**Authors:** Anja Karlstaedt, Fenn Cullen, Rosie Drinkwater, Zenouska Ramchunder, Kyoungmin Kim, Megan Young, Stephen J. Rossiter, Dunja Aksentijevic

**Affiliations:** 1 Department of Cardiology, Smidt Heart Institute, Cedars Sinai Medical Center, Los Angeles, United States of America; 2 William Harvey Research Institute, Barts and the London Faculty of Medicine and Dentistry, Queen Mary University of London, London, United Kingdom; 3 School of Biological and Behaviour Sciences, Queen Mary University of London, London, United Kingdom; 4 Faculty of Veterinary Medicine, Ludwig Maximilian University Munich, Munich, Germany; National Veterinary Research Institute (NVRI), NIGERIA

## Abstract

**Aim:**

The present study aimed to elucidate which pathways contribute to cardiometabolic adaptation in Egyptian fruit bats.

**Methods:**

Utilising cardiac tissues from Egyptian fruit bats (*Rousettus aegyptiacus*) and C57BL/6J mice, we combined liquid chromatography-mass spectrometry metabolic profiling, non-targeted ¹H NMR spectroscopy, and *in silico* computational modelling using the genome-scale mammalian network CardioNet. By integrating complementary untargeted and targeted metabolomics with genome-scale flux balance analysis, this approach enables systems-level inference of pathway activity beyond static metabolite abundance measurements.

**Main findings:**

Our analyses revealed that bat hearts exhibit a distinct metabolic profile characterised by depleted glycogen reserves and increased reliance on lipid oxidation to meet energy demands. Notably, bat hearts displayed elevated fluxes in oxidative phosphorylation, β-oxidation of long-chain fatty acids, and the Krebs cycle, alongside reduced amino acid catabolism. These findings suggest that bats have evolved unique metabolic strategies to support the high-energy demands of flight, maintaining cardiac function without succumbing to pathological remodelling.

**Conclusions:**

This study provides the first comprehensive insight into the metabolic adaptations in the cardiac tissue of a bat species, contributing to our understanding of how these mammals endure extreme physiological stresses.

## Introduction

Bats are exceptional among mammals for sustaining the extreme metabolic demands of powered flight while achieving lifespans far exceeding those of similar-sized non-flying species [1]. Bats also show adaptations in mechanisms underlying DNA repair and inflammatory responses [2–5], and studies conducted on multiple species across several decades have highlighted their capacity for extreme cardiovascular performance under fluctuating energetic demands and resistance to oxidative stress [2,3,6–14].

Flight drives rapid and reversible increases in heart rate and cardiac workload; for example, free-flying bats can exceed 800 beats per minute yet quickly return to low resting rates or even enter torpor under energy-saving conditions [3,12,15]. When resting, many bat species roost in caves or enclosed environments in which microclimatic conditions can be characterized by elevated CO₂ levels and reduced air exchange [16], thus imposing additional respiratory and metabolic challenges. This combination of energetic extremes alongside remarkable longevity makes the bat heart a fascinating model for uncovering metabolic strategies beyond those of canonical mammalian physiology.

Across mammals, mitochondrial metabolic capacity is highly heterogeneous across tissues, reflecting functional specialization in response to differing energetic demands. Comparative studies have shown that mitochondrial biogenesis and oxidative metabolic pathways are differentially regulated across tissues to support specific physiological functions [17–20]. Within this framework, the heart represents one of the most oxidative tissues, requiring continuous ATP production to sustain contractile activity. Several earlier studies have suggested that bat hearts possess unique molecular and physiological traits. For example, comparative genomics reveals alternative splicing of *TNNI3* (cardiac troponin I) isoforms that may facilitate rapid diastolic relaxation at high heart rates without excessive adrenergic stimulation [21]. Mitochondrial studies indicate lower reactive oxygen species production despite high lifetime oxygen fluxes, potentially explaining bats’ resilience to age-related cardiovascular decline [1,6]. Furthermore, frugivorous and nectarivorous bats exhibit dramatic postprandial hyperglycaemia, and stable isotope studies in nectar bats show systemic metabolic switching from lipid oxidation when fasted to carbohydrate oxidation after feeding [22–26]. Yet how these features integrate within the bat myocardium to support high energetic demand without pathological remodelling remains unresolved.

Here, we combine high-resolution metabolomics with *in silico* flux balance analysis to compare the cardiac metabolic networks of the Egyptian fruit bat (*Rousettus aegyptiacus*) and the laboratory mouse. The rationale for our metabolomic and computational approach is to identify systems-level metabolic features that may underpin the extraordinary cardiac performance of the Egyptian fruit bat heart. We hypothesise that the bat heart is metabolically reprogrammed towards enhanced mitochondrial oxidative pathways, particularly long-chain fatty acid β-oxidation and ketone metabolism while maintaining minimal cytosolic energy reserves, enabling sustained ATP production and redox balance under extreme energetic stress. We predict that myocardial metabolomics and flux balance analysis will reveal elevated oxidative phosphorylation metabolic fluxes in bats relative to mice, accompanied by lower cytosolic energy reserve metabolites.

## Materials and methods

### Animals

**Fig 1:**
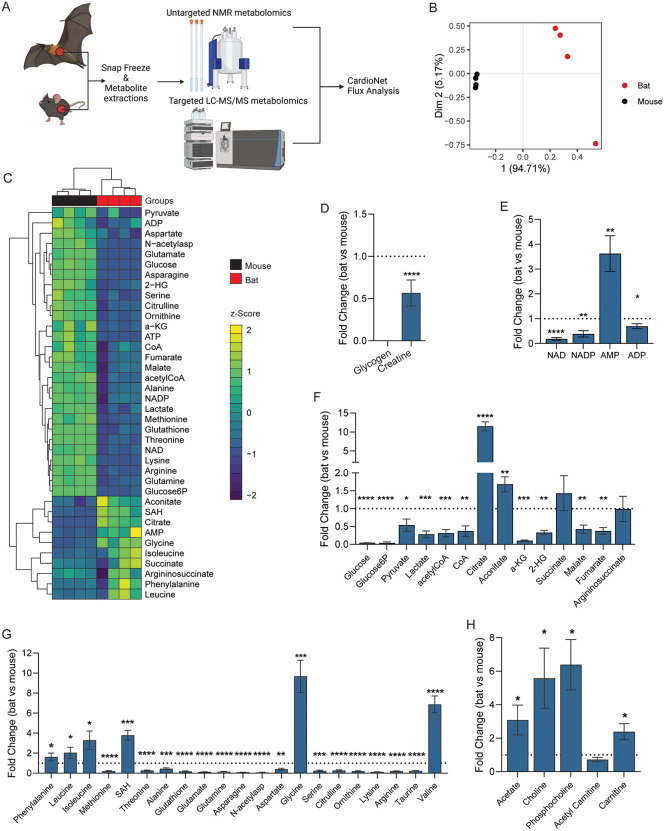
Cardiac metabolism varies between Egyptian fruit bats and C57BL/6J mice. **(A**) Schematic summary of the study protocol. n = 4 bats (n = 2 males, forearm length = 91.60 ± 2.50 mm, n = 2 females, forearm length = 87.90 ± 1.85 mm) and n = 4 C57/BL6J mice (Charles River, UK, male). (**B-C**) Partial Least Squares Discriminant Analysis (PLS-DA) (**B**) and heatmap of unsupervised hierarchical cluster analysis (**C**) of the cardiac metabolite concentrations determined by LC-MS and high-resolution ^1^H nuclear magnetic resonance spectroscopy showing distinct profile of bat and C57/BL6J mouse. (**D-H**) Fold change differences of myocardial intermediates within major metabolic pathways (glucose metabolism, lipid metabolism, Krebs cycle), redox equivalents and energy-providing substrates. Fold changes are expressed as comparisons between bats and C57/BL6J mouse. The normality of data distribution was examined using Shapiro–Wilk’s normality test. All metabolites followed a Gaussian distribution, with the exception of NADP, pyruvate, N-acetylaspartate, and glycine. Comparison between two groups was performed by Student’s t-test (Gaussian data distribution) or Mann-Whitney U test when data was not normally distributed (Panel E, F, and G, NADP, pyruvate, N-acetylaspartate, glycine), *p < 0.05, **p < 0.01, ***p < 0.001, ****p < 0.0001.

We studied bats from a captive population of Egyptian fruit bats (*Rousettus aegyptiacus*, family Pteropodidae) housed at Copenhagen Zoo, Denmark, as previously described [27]. Cardiac tissue samples (n = 4, 2 male, 2 female) were obtained post-mortem from animals euthanized by zoo veterinarians as part of routine population management procedures (Fig 1A). The feeding status of individual bats at the time of sampling could not be controlled or directly assessed.

Twenty-week-old C57BL/6J mice (n = 4, male) were purchased from Charles River, UK (Fig 1A). Mice were housed in individually ventilated cages under controlled temperature (22 ± 2 °C) on a 12:12 h light–dark cycle with ad libitum access to a standard chow diet (irradiated PicoLab® Mouse Diet 20 EXT, 5R58) and water. Mice were euthanized by intraperitoneal phenobarbital injection and hearts were rapidly excised.

### Heart tissue collection

Excised hearts were stabilised in RNAlater and snap-frozen using liquid nitrogen and dry ice. Samples were stored at −80 °C until analysis. Metabolites were extracted from frozen tissue as previously described [3,4].

### Metabolomics

#### ^1^H Untargeted NMR spectroscopy.

Frozen, pulverised cardiac tissue samples (~45 mg) were subject to methanol/water/chloroform phase extraction and analysed using ^1^H NMR high-resolution spectroscopy as previously described [28,29]. ^1^H NMR spectra were acquired using a vertical-bore, ultra-shielded Bruker 14.1. Tesla (600 MHz) spectrometer with a BBO probe at 298K using the Bruker noesygppr1d pulse sequence. Acquisition parameters were 128 scans, 4 dummy scans, and 20.8 ppm sweep width, acquisition time of 2.6s, pre-scan delay of 4s, 90° flip angle, and experiment duration of 14.4 minutes per sample. TopSpin (version 4.0.5) software was used for data acquisition and metabolite quantification. FIDs were multiplied by a line broadening factor of 0.3 Hz and Fourier-transformed, phase and automatic baseline-correction were applied. Chemical shifts were referenced to the TSP signal. Metabolite peaks of interest were initially integrated automatically using a pre-written integration region text file and then manually adjusted where required. Assignment of metabolites to their respective peaks based on previously obtained in-house data, confirmed by chemical shift and using Chenomx NMR Profiler Version 8.1 (Chenomx, Canada). Peak areas were normalised to the total metabolite peak area. Glycogen was quantified using ^¹^H magnetic resonance, measuring the concentration of glucose monomers from normalised peaks. Being a large macromolecule, with possible differences in the mobility of glycosyl units, glycogen has been reported to be fully visible by MRS. The modified dual-phase Folch extraction method, used for separating aqueous and lipid metabolites, was not optimised for extracting glycogen. However, all samples underwent the same extraction procedure, allowing for comparison between groups [28].

### Targeted LC-MS/MS

Frozen pulverised cardiac tissue samples (~15 mg) were extracted using a buffer (50% Methanol, 30% Acetonitrile, 20% ultrapure water, and 50 ng/ml 1 mM HEPES solution) for LC-MS analysis as previously described [30,31]. LC-MS analysis was performed by the metabolic flux analysis facility of the Barts Faculty of Medicine and Dentistry using a Q Exactive Quadrupole-Orbitrap mass spectrometer coupled to a Vanquish UHPLC system (Thermo Fisher Scientific). The liquid chromatography system was fitted with a Sequant ZIC-pHILIC column (150 mm × 2.1 mm) and guard column (20 mm × 2.1 mm) from Merck Millipore (Germany), and the temperature was maintained at 35°C. The sample (3 μL) was separated at a flow rate of 0.1 mL/min. The mobile phase comprised 10 mM ammonium bicarbonate and 0.15% ammonium hydroxide in water (solvent A), and acetonitrile (solvent B). A linear gradient was applied by increasing the concentration of A from 20 to 80% within 22 min and then maintained for 7 minutes. The mass spectrometer was operated in full MS and polarity switching mode, in the range of 70-1000m/z and resolution 70000. Major ESI source settings: spray voltage 3.5kv, capillary temperature 275°C, sheath gas 35, auxiliary gas 5, AGC target 3e6, and maximum injection time 200ms. The acquired spectra were analysed using XCalibur Qual Browser and XCalibur Quan Browser software (Thermo Scientific) for the targeted MS analysis.

### CardioNet-based metabolic flux analysis

*In silico* simulations were conducted using the publicly available metabolic network of the mammalian heart metabolism, CardioNet [32–34]. Mathematical modelling has previously been used to study the dynamics of cardiac metabolism in response to stress. Metabolite abundances from NMR and LC-MS/MS analysis were integrated as constraints for metabolite pools. The objective was to maximize ATP hydrolysis as a reflection of cardiac contraction (vATPase) that satisfies the metabolic constraints. We determined flux distributions (*v*_m_) and estimated flux rate changes (v_FC_) as described previously [29,32,35,36]. We simulated rapid flight, night, and roost activity states using published telemetry heart rate data for the neotropical frugivorous bat *Uroderma bilobatum* [15]. To our knowledge, detailed continuous telemetry datasets are not currently available for *R. aegyptiacus*. We therefore used *U. bilobatum* as an ecologically comparable fruit bat to estimate cardiac ATP demands under different behavioral states. Importantly, the underlying cardiac metabolic data (NMR, LC-MS/MS) used to constrain the model fluxes came directly from *R. aegyptiacus* heart tissue.

Side constraints and parameters for FBA are provided in the Supplementary Material to enable reproducibility and adaptation with new telemetry data as it becomes available. The demand for ATP per heartbeat was based on the transient ATP increase upon excitation [37]. Further, metabolites within the model were constrained based on our bat heart metabolomics analysis results. The uptake of glucose, ketone bodies, amino acids and lipids was not restricted. The GUROBI LP solver was used to find the solution to the FBA problems [38]. These flux distributions represent *in silico* predictions derived from flux balance analysis constrained by metabolomic data, rather than direct measurements of metabolic flux.

### Statistical analysis

Statistical analysis was conducted using GraphPad Prism software (v.10.40.1) and R studio (v.2024.09.0). Differences between groups were considered significant at p < 0.05. Sample sizes were not predetermined based on statistical power calculations. Formal randomizations of mouse or bat experiments were not used. Data were tested for normal distribution and similar variance among treatments using the Shapiro-Wilk tests. For normal distributed metabolomics data, we conducted a t-test followed by multiple-comparison analysis, with a false discovery rate of less than 5% using the two-step method of Benjamini, Krieger, and Yekutieli. For non-normally distributed data, including FBA data, we conducted the Mann-Whitney test followed by multiple comparisons analysis using a false discovery rate of less than 5% using the two-step method of Benjamini, Krieger, and Yekutieli. Unsupervised hierarchical clustering and heatmap visualization were conducted using R package pheatmap (v1.0.12). The following R packages were used for general data analysis: base (v4.3.2), datasets (v4.3.2), tidyverse (v2.0.0); Multivariate Data Analyses and Data Visualization: ggplot2 (v3.5.2), pheatmap (v1.0.12), tidytext (v0.4.2), viridis (v0.6.5.).

### Ethics statement

Bat cardiac tissue was obtained opportunistically from animals euthanized by veterinary staff at Copenhagen Zoo as part of routine population management. No animals were euthanized for the purposes of this study. As these procedures were conducted under the zoo’s established population management practices, no separate research permit or ethics approval was required. Tissue collection was performed post-mortem. All mouse procedures were conducted in accordance with the UK Home Office Animals (Scientific Procedures) Act 1986 under institutional approval.

## Results and discussion

### Results

The analysis of cardiac metabolomics data revealed that Egyptian fruit bats have a distinct cardiometabolic profile compared to C57/BL6J mice (PCA analysis plot, **Fig 1B**). Specifically, the abundance of 43 profiled metabolites significantly differed between bats and mice (**Fig 1C**). Notably, cardiac energy reserves were depleted in bat hearts, as evidenced by reduced creatine and undetectable glycogen (**Fig 1D**). Consistent with these findings, we found increased AMP levels in bat hearts (3.5-fold vs. mouse, **Fig 1E**), while NAD(P)^+^ redox equivalents were reduced. In the bat heart, glycolytic and Krebs cycle intermediates (**Fig 1F**) and amino acids (**Fig 1G**) were reduced compared to mice. Such reductions may reflect tissue-specific regulation of amino acid metabolism, which is known to vary substantially across mammalian tissues according to energetic demand and biosynthetic requirements [39]. Only constituents of lipid metabolism (**Fig 1H**) were universally increased (i.e., acetate, choline, phosphocholine, and carnitine), indicating an increased contribution of lipids to cardiac metabolism in bats. Correlation analysis of metabolic intermediates (**S1 Fig**) revealed co-regulation of ketogenic amino acids entering the Krebs cycle through acetyl-CoA. Increased ketone body metabolism spares protein degradation and further supports lipid oxidation. Together, our metabolomics findings suggest metabolic adaptation in the bats characterized by increased oxidation of lipids to meet cardiac energy demands.

**Fig 2:**
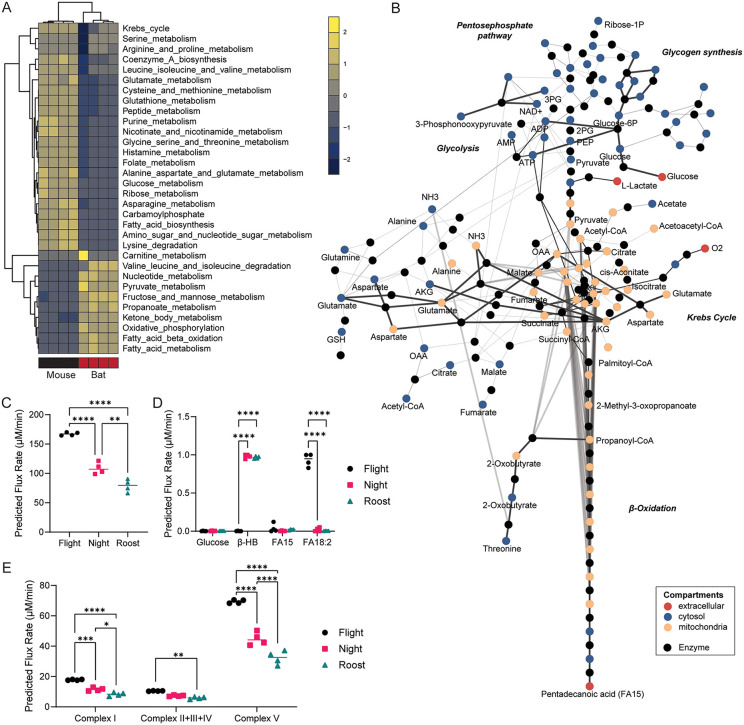
CardioNet analysis reveals increased lipid oxidation flux in bats. (**A**) Unsupervised hierarchical clustering of estimated z-scored flux rate changes reveals metabolic adaptation in bats vs mice. Z-scores were calculated to visualise how many standard deviations an estimated flux rate is away from the mean across all experimental groups. The z-score describes the distance from the mean for a given flux rate as a function of the standard deviation. The colour scale indicates the degree to which estimated flux rate changes are predicted to be respectively lower or higher bat vs mouse. (**B**) CardioNet *in silico* metabolic >5-fold flux changes bat versus C57/BL6J. The graph denotes the estimated flux distribution. The coloured nodes represent metabolites assigned to five compartments: extracellular space, cytosol, mitochondria, microsome, and lysosome. The black nodes indicate reactions; two reactions are linked by a directed edge, indicating the reaction flux. The line thickness of each edge is proportional to the predicted flux rate change. (**C**) Predicted myocardial oxygen consumption in simulations of bat flight, night activity and roost activity (n = 4/group). (**D**) Predicted substrate uptakes for glucose, β-hydroxybutrate (β-HB) and fatty acids (FA15, FA18:2) in simulations of bat flight, night activity and roost activity (n = 4/group). (**E**) Predicted flux rates for oxidative phosphorylation and electron transport chain in simulations of bat flight, night activity and roost activity (n = 4/group).

Our systems biology approach revealed that bat hearts show a manifold increase in cardiac metabolic flux of oxidative phosphorylation, β-oxidation of long-chain fatty acids, glutamate, pyruvate, Krebs cycle (α-ketoglutarate), and total adenine nucleotide pool (AMP, ADP, ATP; **Fig 2A**). Furthermore, bat hearts were characterised by the markedly elevated flux of fructose, ketone body metabolism, and reduction in amino acid catabolic flux exchange (**Fig 2B**).

Comparison of CardioNet-simulated predicted flux distributions from different activity states in bats (night, roost, and flight) show different cardiac metabolic profiles (**S2 Fig**). Predicted myocardial oxygen consumption increased with activity status (**Fig 2C**), with flight showing the highest consumption rate, followed by night and roost activity. These differences were also reflected in the consumption of energy-providing nutrients. The glucose utilisation is predicted to be low in all three activity states (**Fig 2D**). The uptake and oxidation of linoleate (FA18:2) is highest during flight, while β-hydroxybutyrate contribution increases significantly during roost and night activity. This difference was also reflected at the level of the mitochondrial electron transport chain fluxes (**Fig 2E**), where complex I and V fluxes increase significantly during flight and are lowest during roost activity. Thus, fatty acids are major energy-providing substrates, and ketone bodies further complement ATP provision during roost and night activities.

## Discussion

Our study provides the first integrative metabolomic and flux balance analysis of Egyptian fruit bat (*Rousettus aegyptiacus*) myocardium, revealing distinct metabolic signatures compared to the widely used murine model (C57/BL6). By combining untargeted and targeted metabolomics with *in silico* modeling constrained by empirical data, we identify key pathways, particularly long-chain fatty acid and ketone body oxidation, that support the extreme energetic demands of the bat heart while minimizing reliance on carbohydrate reserves.

Classic physiological studies established that bats maintain exceptional cardiac performance during energetically costly flight [14,40]. Our findings align with this literature while extending it by identifying metabolic pathways that may underlie this performance. Our data suggest that, in bat hearts, glycogen stores are depleted and that lipid oxidation is the dominant energy-providing substrate. This observation is consistent with broader evidence that energetically demanding tissues selectively upregulate lipid metabolic pathways to support sustained oxidative ATP production [41].

In the heart, glycogen reserves are normally utilised during periods of high physical activity [42] or a means of adaptation to extreme physiological environments, such as was recently observed in naked mole rats [28]. High AMP concentration signals a highly catabolic cellular state and allosterically inhibits phosphofructokinase in glycolysis, which supports replenishing glycogen pools [36]. Our cardiac metabolic profile results align with isotopic tracer studies of systemic metabolism demonstrating that *R. aegyptiacus* rapidly and directly oxidizes exogenously supplied substrates to fuel both rest and flight [43]. Simulation of activity states revealed that the bat heart shifts from predominantly oxidizing fatty acids (FA18:2) to ketone bodies (β-hydroxybutyrate) during lower activity states at night and during roosting. Interestingly, myocardial ketone body and branched-chain amino acid oxidation exceed glucose oxidation *in silico*, which requires further experimental validation. While the frugivorous diet of *R. aegyptiacus* is relatively protein-poor, our model predictions of greater amino acid oxidation in cardiac tissue likely reflect the endogenous catabolism of intracellular amino acid pools, rather than a reliance on dietary amino acids *per se*.

Our previous work [25] in multiple non-cardiac tissues demonstrated positive selection in carbohydrate metabolism genes across nectar- and fruit-feeding bats, consistent with a high capacity for carbohydrate flux. In our study, the *R. aegyptiacus* heart showed high predicted flux through oxidative pathways, including fatty acid oxidation, at the time of sampling. These findings are not mutually exclusive: while the bat heart may retain the capacity for rapid carbohydrate oxidation under fed or exercise conditions, cardiac energy supply during rest or fasting may rely predominantly on fatty acid oxidation. *R. aegyptiacus* has been shown to exhibit dramatic postprandial hyperglycaemia, which would affect myocardial exogenous substrate supply [22,44]. Our tissue samples were obtained opportunistically and thus could not control for feeding status or recent activity. Future studies combining plasma and tissue metabolomics under controlled fasted, fed, and exercise conditions will be crucial to capture the full dynamic range of cardiac fuel utilisation.

A key advance of this study is integrating metabolite concentrations with flux balance analysis (CardioNet) to estimate pathway-level metabolic activity rather than relying solely on static metabolite abundances. This approach enables generation of mechanistic hypotheses; for example, that fatty acid and ketone oxidation dominate basal myocardial energy supply in *R. aegyptiacus*. Nevertheless, we acknowledge that flux predictions require experimental validation, ideally via isotope tracing or perfused heart studies under controlled workloads and nutritional states.

We explicitly acknowledge several limitations of this study. First, the analysis is based on a two-species comparison, and therefore the results should be interpreted as exploratory rather than as broad evolutionary generalisations. The C57BL/6J mouse serves here as a physiologically tractable reference rather than a phylogenetic control, enabling identification of metabolic pathways potentially associated with the exceptional energetic resilience of the bat heart. Broader comparative studies across multiple bat and non-bat species will be required to distinguish lineage-specific adaptations from general mammalian variation. Bat samples included both males and females, whereas mouse samples were male-only; this imbalance may represent a potential confounder and should be considered when interpreting interspecies comparisons.

Second, comparative metabolic research in non-model mammals, particularly bats, is inherently constrained by ethical and conservation considerations. Bat species reproduce slowly, and many are protected in the wild, meaning that opportunities to obtain cardiac tissue are extremely limited. Although our sample size is modest, metabolite profiles were consistent across individuals, supporting the robustness of the main metabolic patterns observed.

Third, the study captures only a single metabolic state. Feeding status and recent activity could not be controlled because cardiac tissue was obtained opportunistically during routine zoological population management. Given the well-known metabolic flexibility of frugivorous bats, the myocardial profile described here represents a snapshot of the physiological state at the time of sampling. Nevertheless, the metabolic phenotype observed was internally coherent, with coordinated changes in glycogen, lipid-associated metabolites, redox balance, and model-predicted oxidative fluxes, suggesting a myocardial metabolic configuration consistent with high oxidative capacity under the sampled condition.

Finally, the computational modelling has inherent limitations. CardioNet was originally parameterised using biochemical knowledge derived primarily from human and murine systems, and species-specific differences in enzyme kinetics, transport processes, and regulatory mechanisms may influence predicted flux distributions when applied to non-model species. Accordingly, the simulations presented here should be interpreted as metabolomics-constrained, hypothesis-generating predictions rather than direct measurements of metabolic flux. In addition, cardiac ATP demand in the simulations was parameterised using heart rate telemetry data from *Uroderma bilobatum*, as continuous telemetry datasets are not currently available for *R. aegyptiacus*. Although both are frugivorous bats with comparable ecological niches, interspecific differences in body mass and flight behaviour may influence quantitative ATP demand estimates [10,11,14,15]. Importantly, however, the metabolomic constraints used to parameterise flux distributions were derived directly from *R. aegyptiacus* myocardium, ensuring that pathway-level predictions reflect the measured biochemical state of the studied species.

Despite limitations, our study offers rare and valuable insights into bat cardiac metabolism, highlighting a shift toward oxidative substrates and minimal cytosolic energy reserves. We propose that metabolic flexibility switching between carbohydrate, lipid, and ketone oxidation may underpin the bat heart’s ability to sustain extreme energetic demands without pathological remodelling. Future work should prioritize controlled feeding and exercise experiments with paired plasma-tissue metabolomics, isotope tracing to validate flux predictions and multi-species comparisons to dissect ecological versus phylogenetic influences on cardiac metabolism. Such studies will clarify whether the pathways highlighted here represent general principles of chiropteran cardiac physiology or unique adaptations of *R. aegyptiacus*.

### Conclusion

This study provides the first integrative metabolomic and genome-scale modelling analysis of cardiac metabolism in the Egyptian fruit bat (*Rousettus aegyptiacus*). By combining untargeted and targeted metabolomics with metabolomics-constrained flux balance analysis, we identify a myocardial metabolic configuration characterised by depleted glycogen reserves and increased reliance on mitochondrial oxidative pathways, particularly fatty acid and ketone body oxidation. These findings suggest that the bat heart sustains energetic demand through dominant oxidative metabolism while maintaining minimal cytosolic energy reserves. Although the modelling results capture metabolic profiles from bat heart tissue samples, several predictions require additional experimental validation. Nonetheless, this work provides rare systems-level insight into cardiac metabolism in a non-model mammal and establishes a framework for future comparative and experimental studies aimed at understanding metabolic strategies supporting extreme cardiovascular performance.

## Supporting information

S1 Fig Correlation analysis of bat metabolome. Metabolite abundances from NMR-and LC-MS/MS-based metabolomics were analysed using Spearman correlation to reveal interactions between intermediates. Positively and negatively correlating intermediates are indicated by blue and red colour coding, respectively. Circle sizes indicate calculated absolute spearman coefficient ranging from 0 to 1.
(TIF)

S2 Fig *In silico* modelling of bat metabolism. (A) Unsupervised hierarchical cluster analysis and heatmap of significantly altered metabolic fluxes in bat and mouse simulations using CardioNet. Flux rates were z-score normalised. n = 4/species. (B) Principal component analysis of simulated activity states: flight, night and roost.
(TIF)

S1 File Metabolomic and ^1^H nuclear magnetic resonance spectroscopy data. (XLSX)

S2 File Metabolomic and activity constraints data. (XLSX)
