# Supplementary figures and images for: Metabolomics-constrained modelling reveals dominant oxidative metabolism in the Egyptian fruit bat myocardium
Source: PLoS One. 2026 Jun 2;21(6):e0349571. doi: 10.1371/journal.pone.0349571 (PMC13229291; doi:10.1371/journal.pone.0349571)

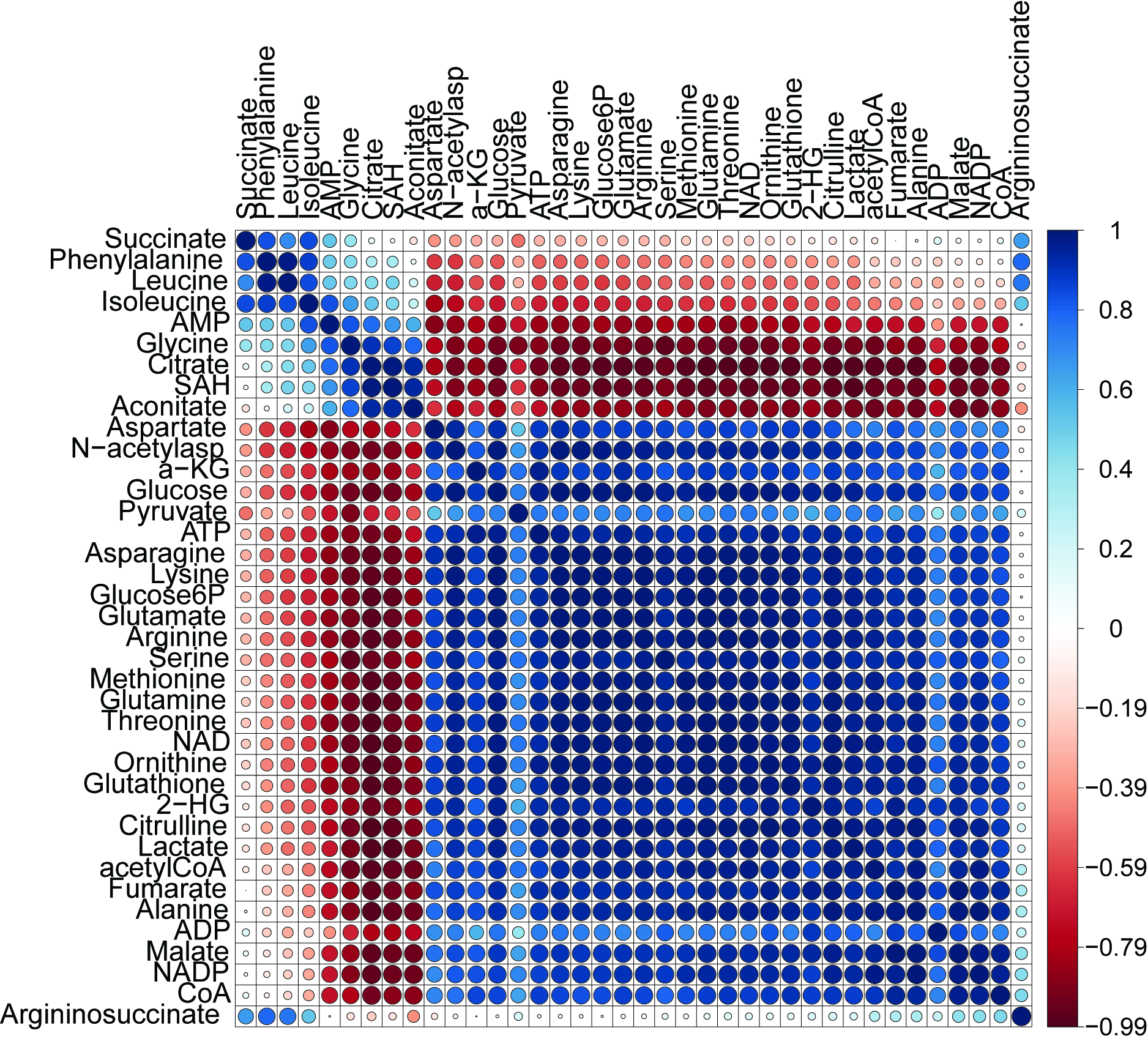

Supplement: S1 Fig — Metabolite abundances from NMR-and LC-MS/MS-based metabolomics were analysed using Spearman correlation to reveal interactions between intermediates. Positively and negatively correlating intermediates are indicated by blue and red colour coding, respectively. Circle sizes indicate calculated absolute spearman coefficient ranging from 0 to 1. (TIF) [file pone.0349571.s001.tif]

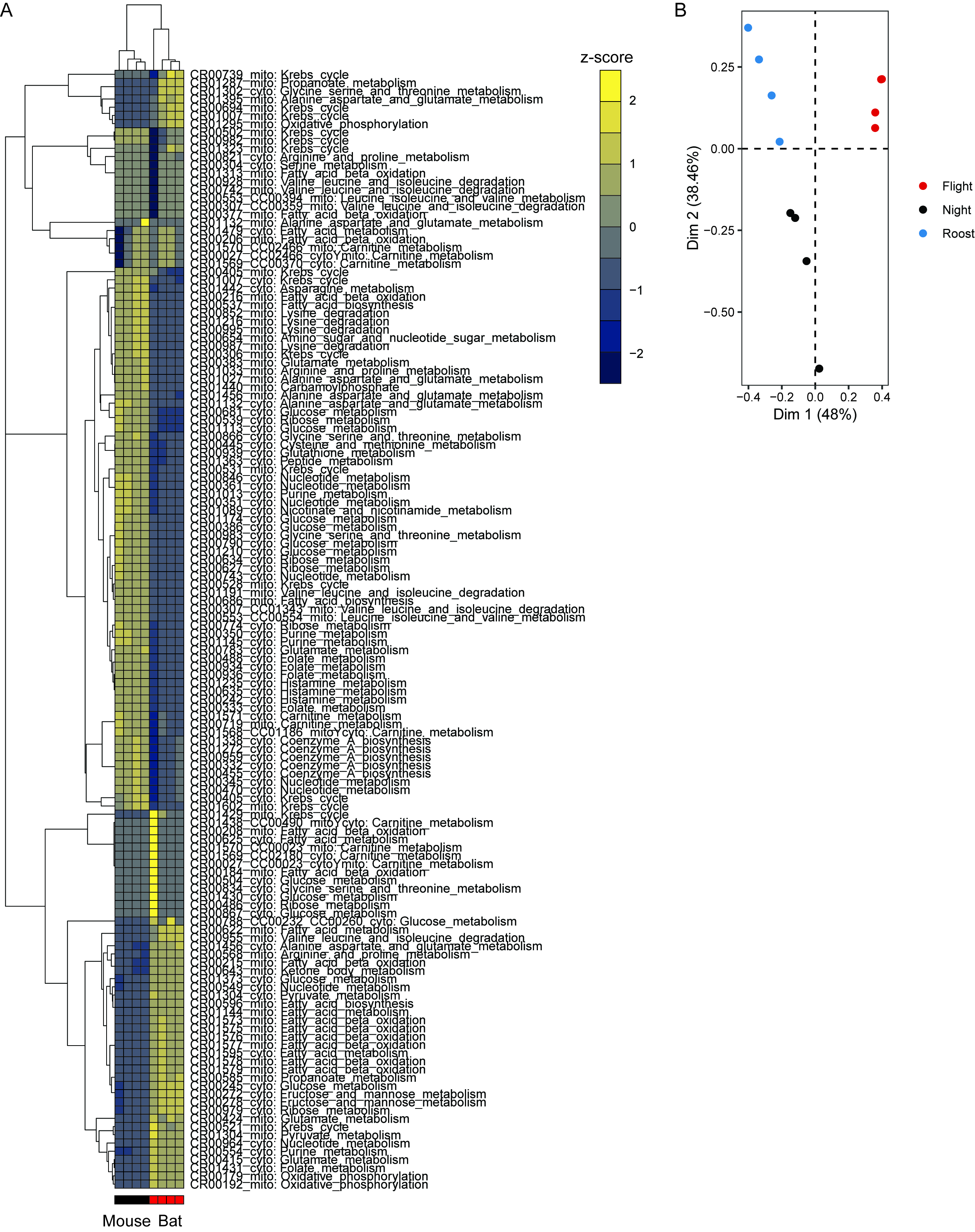

Supplement: S2 Fig — (A) Unsupervised hierarchical cluster analysis and heatmap of significantly altered metabolic fluxes in bat and mouse simulations using CardioNet. Flux rates were z-score normalised. n = 4/species. (B) Principal component analysis of simulated activity states: flight, night and roost. (TIF) [file pone.0349571.s002.tif]
